# Shedding Light on Microbial “Dark Matter”: Insights Into Novel Cloacimonadota and Omnitrophota From an Antarctic Lake

**DOI:** 10.3389/fmicb.2021.741077

**Published:** 2021-10-11

**Authors:** Timothy J. Williams, Michelle A. Allen, Jonathan F. Berengut, Ricardo Cavicchioli

**Affiliations:** ^1^School of Biotechnology and Biomolecular Sciences, UNSW Sydney, Sydney, NSW, Australia; ^2^EMBL Australia Node for Single Molecule Science, School of Medical Sciences, UNSW Sydney, Kensington, NSW, Australia

**Keywords:** Cloacimonadota, Omnitrophota, cellulosome, autotrophy, metagenome, Antarctic bacteria

## Abstract

The potential metabolism and ecological roles of many microbial taxa remain unknown because insufficient genomic data are available to assess their functional potential. Two such microbial “dark matter” taxa are the *Candidatus* bacterial phyla Cloacimonadota and Omnitrophota, both of which have been identified in global anoxic environments, including (but not limited to) organic-carbon-rich lakes. Using 24 metagenome-assembled genomes (MAGs) obtained from an Antarctic lake (Ace Lake, Vestfold Hills), novel lineages and novel metabolic traits were identified for both phyla. The Cloacimonadota MAGs exhibited a capacity for carbon fixation using the reverse tricarboxylic acid cycle driven by oxidation of hydrogen and sulfur. Certain Cloacimonadota MAGs encoded proteins that possess dockerin and cohesin domains, which is consistent with the assembly of extracellular cellulosome-like structures that are used for degradation of polypeptides and polysaccharides. The Omnitrophota MAGs represented phylogenetically diverse taxa that were predicted to possess a strong biosynthetic capacity for amino acids, nucleosides, fatty acids, and essential cofactors. All of the Omnitrophota were inferred to be obligate fermentative heterotrophs that utilize a relatively narrow range of organic compounds, have an incomplete tricarboxylic acid cycle, and possess a single hydrogenase gene important for achieving redox balance in the cell. We reason that both Cloacimonadota and Omnitrophota form metabolic interactions with hydrogen-consuming partners (methanogens and Desulfobacterota, respectively) and, therefore, occupy specific niches in Ace Lake.

## Introduction

Microorganisms make up the majority of the biomass of the planet, yet the genomic potential of many microbial species remains elusive. The existence of many hitherto unknown taxa has only been revealed through cultivation-independent approaches, particularly from 16S rRNA gene libraries, and metagenome data and the analysis of metagenome-assembled genomes (MAGs) (Hugenholtz et al., 1998; Rinke et al., 2013; Momper et al., 2017; Parks et al., 2017, 2020; Nayfach et al., 2020; Zamkovaya et al., 2021). Uncultivated clades, referred to as “microbial dark matter,” include lineages that are inferred to play key roles in ecosystem formation and nutrient cycling (Rinke et al., 2013; Parks et al., 2017, 2020; Nayfach et al., 2020; Zamkovaya et al., 2021), including in Antarctica (Cavicchioli, 2015; Panwar et al., 2020). The phyla *Candidatus* Cloacimonadota and *Candidatus* Omnitrophota (hereafter Cloacimonadota and Omnitrophota, respectively) are inferred to contribute to anaerobic recycling of organic matter, although their ecophysiological traits remain largely undetermined (Baricz et al., 2020; Suominen et al., 2021).

Phylum Cloacimonadota [originally WWE1 (“Waste Water of Evry 1”); Chouari et al., 2005a,b] belongs to the “Fibrobacteres-Chlorobia-Bacteroidetes” (FCB) superphylum of bacteria (Rinke et al., 2013). Cloacimonadota can be a major component of anaerobic digestors and especially important in lipid-rich waste (Toth and Gieg, 2018; Saha et al., 2019; Shakeri Yekta et al., 2019). The first named member of this phylum, *Ca.* Cloacimonas acidaminovorans, is based on a MAG from an anaerobic digester of a municipal wastewater treatment plant (Chouari et al., 2005a,b; Pelletier et al., 2008). Based on single-cell amplified genome (SAG) and metatranscriptomic analyses, syntrophic propionate oxidation was inferred for a novel *Ca.* Cloacimonas species from a terephthalate-degrading bioreactor (Nobu et al., 2015) and for *Ca.* Syntrophosphaera thermopropionivorans based on a MAG from a thermophilic biogas reactor (Dyksma and Gallert, 2019). As well as being recovered from anaerobic digesters, abundant Cloacimonadota have also been detected in natural environments, including in anoxic and sulfidic water layers of the Black Sea (Suominen et al., 2021; Villanueva et al., 2021), Ursu Lake, Romania (Baricz et al., 2020), and from the Thuwal cold seep brine pool of the Red Sea (Zhang et al., 2016). Based on incubations with complex carbon substrates and analysis of MAGs, the Black Sea Cloacimonadota were inferred to be fermentative heterotrophic generalists capable of assimilating diverse carbon sources, including proteins (Suominen et al., 2021).

Phylum Omnitrophota [originally candidate division OP3 (Obsidian Pool 3)] was first discovered in 16S rRNA gene libraries generated from a hot spring sediment at the Yellowstone National Park (Hugenholtz et al., 1998). Further Omnitrophota sequences were detected in anoxic environments such as terrestrial subsurface fluids, flooded paddy soils, marine sediments, lagoon sediments, hypersaline deep sea waters, freshwater lakes, aquifers, methanogenic bioreactors, and acidic peatland soils (Derakshani et al., 2001; Glöckner et al., 2010; Rinke et al., 2013; Dombrowski et al., 2017; Momper et al., 2017; Lin et al., 2020; Santos et al., 2020). Phylogenetically, Omnitrophota has been assigned to the “Planctomycetes-Verrucomicrobia-Chlamydiae” superphylum based on 16S rRNA analysis (Wagner and Horn, 2006; Pilhofer et al., 2008; Glöckner et al., 2010), which was confirmed using metagenome-based analysis (Rinke et al., 2013). The nominative species *Candidatus* Omnitrophus fodinae SCGC AAA011-A17 is based on a SAG from groundwater (Homestake Mine, South Dakota); genome analysis of this SAG, and other MAGs from the same deep subsurface locality, indicated capacities for carbon fixation by the Wood–Ljungdahl (WL) pathway (reductive acetyl-CoA pathway) (Rinke et al., 2013; Momper et al., 2017). The latter MAGs also possessed genes for hydrogen (H_2_) oxidation, methane oxidation, and dissimilatory nitrate reduction (Momper et al., 2017). Single-cell analysis of *Ca.* Omnitrophus magneticus SKK-01 isolated from the suboxic layer of lake sediments (Lake Chiemsee, Bavaria) revealed ovoid, flagellated cells that harbored intracellular sulfur inclusions and chains of magnetite (Kolinko et al., 2012); analysis of genome sequences identified genes associated with magnetosome biosynthesis, sulfur oxidation, and carbon fixation (Kolinko et al., 2016). Genomic analysis of an Omnitrophota MAG (“bin146”) from the Black Sea inferred a fermentative heterotroph that scavenged low-molecular-weight organic substrates and was capable of glycolysis to acetate as well as H_2_ production (Suominen et al., 2021).

Both Cloacimonadota and Omnitrophota were detected in Ace Lake (Panwar et al., 2020), a marine-derived, meromictic (stratified) system in the Vestfold Hills of Antarctica (Rankin et al., 1999; Lauro et al., 2011). The interface of the lake (12–15 m) is defined by a strong halocline and oxycline, dominated in the austral summer months by a species of the green sulfur bacterium *Chlorobium* (Ng et al., 2010; Lauro et al., 2011; Panwar et al., 2020). The oxic–anoxic interface and lower anoxic zone (16–24 m) support anaerobes, including members of Cloacimonadota and Omnitrophota, which were among the most abundant taxa, with peak relative abundances of 16 and 5%, respectively (Panwar et al., 2020). Preliminary analysis of the Ace Lake Cloacimonadota MAGs inferred a chemolithoautotrophic carbon fixation capacity driven by H_2_ oxidation, while the functional potential of the Ace Lake Omnitrophota MAGs was not examined (Panwar et al., 2020). The Ace Lake data represents 120 metagenomes generated from size-fractionated samples representing a depth profile and a 10-year sampling period (Panwar et al., 2020). The large metagenome dataset provided a unique opportunity to reconstruct the metabolisms of these two “dark matter” candidate phyla, infer their ecophysiology, and consider the potential ecological niches they occupy in Ace Lake.

## Materials and Methods

Microbial biomass was sampled from Ace Lake in austral summers of 2006/2007 and 2008/2009, and a full Antarctic seasonal cycle of summer 2013/2014 to summer 2014/2015. Biomass was collected by sequential size fractionation through a 20 μm prefilter onto 3.0, 0.8-, and 0.1-μm pore-sized, large format (293-mm polyethersulfone membrane) filters, and DNA was extracted from the biomass as described previously (Ng et al., 2010). Six depths were sampled (surface, 5, 11.5–13, 12.7–14.5, 14–16, 18–19, and 23–24 m) with the precise depths varying depending on the water level in the lake (Panwar et al., 2020). In winter 2014, samples were not taken below the oxic–anoxic interface (Panwar et al., 2020). DNA was sequenced and the sequences uploaded to Integrated Microbial Genomes (IMG) (Huntemann et al., 2015) generating 120 individual metagenomes, as described previously (Panwar et al., 2020). High- and medium-quality MAGs were auto-generated from individual metagenomes during the IMG pipeline process. QC-filtered raw reads from the individual Ace Lake metagenomes were co-assembled using Megahit v1.1.1 (Li et al., 2016) with a setting of meta-large, and MAGs were generated from the co-assembly using MetaBAT v2.12.1 with minContig length 2,500 bp (Kang et al., 2019). MAGs from the co-assembly (available in IMG as Metagenome ID 3300035698) were assessed for completeness and contamination using CheckM v1.0.7 (Parks et al., 2015), for taxonomic identity using RefineM v 0.0.23 (Parks et al., 2017), and for phylogenetic placement using Genome Taxonomy Database Toolkit (GTDB-Tk) v.1.4.0 with GTDB release R95 (Chaumeil et al., 2019; Parks et al., 2020). The GTDB-Tk dependencies were pplacer (Matsen et al., 2010), FastANI (Jain et al., 2019), Prodigal (Hyatt et al., 2010), FastTree 2 (Price et al., 2010), HMM (Eddy, 2011), and Mash (Ondov et al., 2016).

Metagenome-assembled genomes from the individual Ace Lake metagenomes and from the co-assembly that belonged to the phyla Cloacimonadota (one high- and 21 medium-quality MAGs) and Omnitrophota (9 high- and 72 medium-quality MAGs) were grouped based on average nucleotide identity (FastANI v 1.32; Jain et al., 2019), and average amino acid identity (CompareM v 0.1.1^1^) to identify representative MAGs for further examination (Supplementary Table S1).

Phylogenetic trees showing all Cloacimonadota and Omitrophota MAGs were generated by GTDB-Tk, based on a ∼5,000 amino acid-long concatenated multiple sequence alignment of 120 bacterial reference genes, and viewed with Dendroscope 3.5.7 (Huson and Scornavacca, 2012). Maximum likelihood phylogenies of selected novel and reference Cloacimonadota and Omnitrophota taxa were generated from the same GTDB-Tk concatenated multiple sequence alignments using W-IQ-Tree (Nguyen et al., 2015; Trifinopoulos et al., 2016) with autoselection of the best-fit model and 1,000 ultrafast bootstraps (Minh et al., 2013).

Of the total of 22 Cloacimonadota and 81 Omnitrophota MAGs identified in Ace Lake, certain MAGs were chosen for in-depth genomic examination, based on the aim of sampling the total known phylogenetic diversity of the respective phyla, as well as completeness of the MAGs (Supplementary Table S1). On this basis, 10 Cloacimonadota MAGs and 14 Omnitrophota MAGs were chosen. The genomic functional potential of the MAGs was assessed by considering cellular and metabolic traits based upon manual examination of proteins and pathways that was performed in a similar way to previous assessments of the veracity of gene functional assignments (Allen et al., 2009; Panwar et al., 2020; Williams et al., 2021). This method included the vetting via manual curation of the IMG protein annotations used in this study. All protein sequences were submitted to ExPASy BLAST (using the “UniProtKB/Swiss-Prot only” option) (Gasteiger et al., 2003); proteins needed to show ≥ 35% sequence identity to an experimentally verified protein in the ExPASy BLAST database for the functional annotation to be considered valid. If this threshold was not reached, protein sequences were submitted to InterProScan (Blum et al., 2020) to identify functional domains (e.g., catalytic domains; dockerin and cohesin domains) and potential subcellular locations (e.g., using signal peptides for an extracytoplasmic location; transmembrane helices for a membrane location). IMG annotations that could not be verified using this process were discarded. All of our protein identifications are considered putative. GH families were identified according to the CAZy (Carbohydrate-Active enZymes) classification (Lombard et al., 2014). Protein sequences that were identified as hydrogenases based on catalytic domains were classified further using the hydrogenase classifier HydDB (Søndergaard et al., 2016). Only those MAGs that were subjected to in-depth examination are named here; these were named according to recommendations for describing novel *Candidatus* species (Konstantinidis et al., 2017; Chuvochina et al., 2019; Murray et al., 2020).

## Results and Discussion

### Genomic Assemblies and Phylogenetic Analysis

**FIGURE 1:**
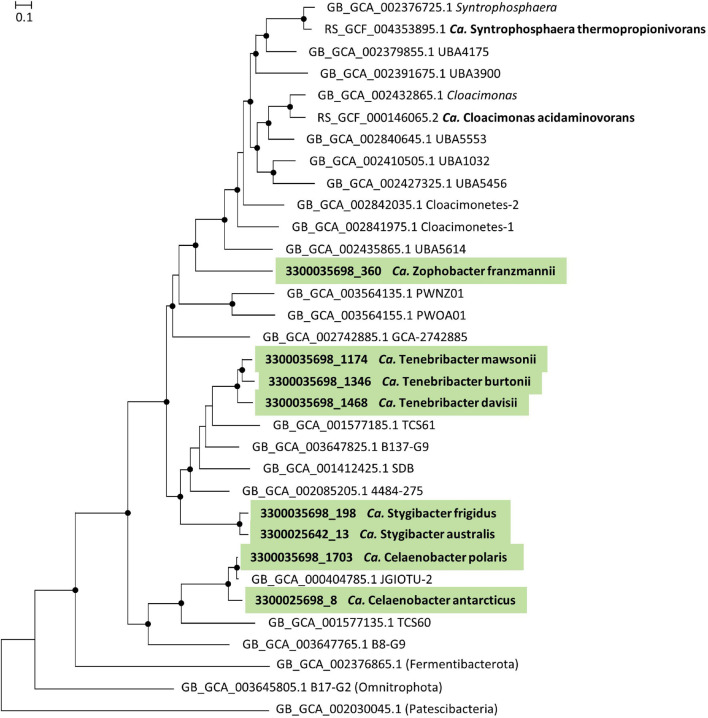
Phylogeny of phylum *Candidatus* Cloacimonadota. Maximum likelihood tree constructed in IQ-Tree with autoselection of the best-fit model (LG + F + I + G4) and 1,000 ultra-fast bootstraps. UFBootstraps ≥ 95% (black dot); Metagenome-assembled genomes (MAGs) featured in this study (green) with their IMG MAG ID and proposed *Candidatus* genus and species name. Reference Cloacimonadota MAGs are shown with their Genome Taxonomy Database (GTDB) accession and GTDB taxonomy, except for *Candidatus* Cloacimonas acidaminovorans and *Candidatus* Syntrophosphaera thermopropionivorans, which already have names. The tree is rooted using a representative of the Patescibacteria.

The genomes of 10 Cloacimonadota MAGs were interrogated (58–97% completeness; 0–4.4% contamination), which represent four novel genus-level and eight novel species-level taxa (Table 1 and Supplementary Table S2). Based on phylogenetic analysis and GTDB taxonomy, the four novel genera are deeply nested within the phylum Cloacimonadota, within the class *Candidatus* Cloacimonadia. None of the four genera were found to be closely related to *Ca.* Cloacimonas or *Ca.* Syntrophosphaera, both of which belong to the family *Candidatus* Cloacimonadaceae (Dyksma and Gallert, 2019; Figure 1 and Supplementary Figure S1).

**TABLE 1:** Metabolic capacity of the Ace Lake Cloacimonadota inferred from metagenome-assembled genomes (MAGs).

Cloacimonadota		Metabolic capacity						
All genera and species		Anaerobic heterotroph secreted glycoside hydrolases and peptidases F-type ATP synthase						
*Candidatus* Genus	*Candidatus* Species	MAGs (% completeness)	Extracellular features + secreted GHs	Fermentation substrates	Carbon fixation	Sulfur metabolism	Other bioenergetic complexes/enzymes	Hydrogenases
Tenebribacter	burtonii^T^ davisii mawsonii	3300035698_1346 (97%) 3300035698_1468 (97%) 3300035698_1174 (91%)	Poly-γ-glutamate synthesis; β-glucanase, β-glucosidase, glucosylceramidase, chitinase	Sugars, amino acids, 2-oxoacids, aldehydes, alcohols, glycerol, formate	Reverse tricarboxylic acid cycle	Oxidation of sulfur compounds (including thiosulfate), linked to Hdr reduction	Rnf, Nqr, Sud, HppA	Membrane-bound, H_2_-evolving NiFe hydrogenase (Group 4g) linked to Mrp; FeFe hydrogenase (Group C1) for redox balance
Stygibacter	australis^T^ frigidus	3300025642_13 (88%), 3300035698_2003 (69%) 3300035698_198 (58%)	Poly-γ-glutamate synthesis; halomucin-like protein; cellulosome-like; β-glucanase, β-glucosidase, α-amylase, glucosylceramidase, chitinase	Sugars, amino acids, 2-oxoacids, aldehydes, alcohols, glycerol, formate	Reverse tricarboxylic acid cycle	Oxidation of sulfur compounds (including thiosulfate), linked to Hdr reduction	Rnf, Nqr, Sud, HppA	Membrane-bound, H_2_-evolving NiFe hydrogenase (Group 4g) linked to Mrp; FeFe hydrogenases (Groups A3 and C1) for redox balance
Zophobacter	franzmannii ^T^	3300035698_360 (80%)	Poly-γ-glutamate synthesis; β-glucanase, β-glucosidase, chitinase	Sugars, amino acids, 2-oxoacids, aldehydes, glycerol, formate	–	–	Rnf, Nqr, Sud, HppA	–
Celaenobacter	antarcticus ^T^ polaris	3300025698_8 (92%), 3300035698_1683 (65%) 3300035698_1703 (91%)	β-glucosidase, β-galactosidase	Sugars, amino acids, 2-oxoacids, aldehydes, formate	Reverse tricarboxylic acid cycle	Assimilatory sulfate reduction	Rnf, Nqr, Sud, HppA	Membrane-bound, NiFe H_2_-evolving hydrogenase (Group 4g) linked to Mrp; H_2_-oxidizing, Hdr-linked cytoplasmic NiFe hydrogenase (Group 3c)

*GH, glycoside hydrolase; Hdr, heterodisulfide reductase; HppA, pyrophosphate-energized sodium pump; Mrp, multicomponent Na^+^:H^+^ antiporter; Nqr, sodium-translocating NADH:quinone oxidoreductase; Rnf, ferredoxin:NAD^+^-oxidoreductase complex; Sud, bifunctional sulfide dehydrogenase/ferredoxin:NADP oxidoreductase. ^T^Indicates type species.*

**FIGURE 2:**
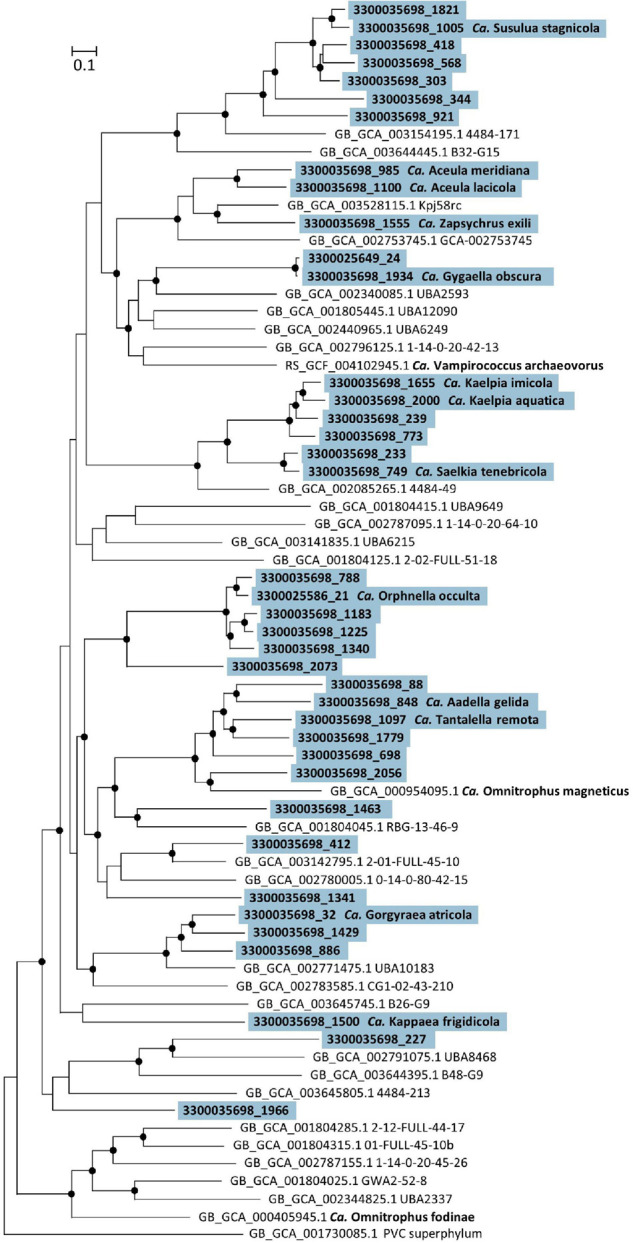
Phylogeny of phylum *Candidatus* Omnitrophota. Maximum likelihood tree constructed in IQ-Tree with autoselection of the best-fit model (LG + F + I + G4) and 1,000 ultra-fast bootstraps. UFBootstraps ≥ 95% (black dot); Metagenome-assembled genomes (MAGs) featured in this study (blue) with their IMG MAG ID and proposed *Candidatus* genus and species names. Reference Omnitrophota MAGs are shown with their Genome Taxonomy Database (GTDB) accession and GTDB taxonomy, except for *Candidatus* Omnitrophus fodinae and *Candidatus* Omnitrophus magneticus, which already have names. The tree was rooted with a basal representative of the “Planctomycetes-Verrucomicrobia-Chlamydiae” (PVC) superphylum.

The genomes of 14 Omnitrophota MAGs were interrogated (61–93% completeness; 0–9.1% contamination) that represent 11 novel genus-level and 13 novel species-level taxa (Table 2 and Supplementary Table S2). Phylogenetic analysis and GTDB taxonomy revealed that the 11 genera represent two class-level and eight order-level clades. None of the Ace Lake MAGs were closely related to *Ca.* Omnitrophus (class *Candidatus* Omnitrophia, order *Candidatus* Omnitrophales), which the phylogenetic analysis recovered in a relatively basal position within the phylum (Figure 2 and Supplementary Figure S2).

**TABLE 2:** Metabolic capacity of the Ace Lake Omnitrophota inferred from metagenome-assembled genomes (MAGs).

Omnitrophota		Metabolic capacity				
All genera and species		Anaerobic heterotroph incomplete TCA cycle, terminating at fumarate (reductive branch) and 2-oxoglutarate (oxidative branch) secreted glycoside hydrolases and peptidases V-type ATP synthase				
	
*Candidatus* Genus	*Candidatus* Species	MAGs (% completeness)	Fermentation substrates	Other carbon metabolism	Other bioenergetic complexes/enzymes	Hydrogenase
Aceula	lacicola^T^	3300035698_1100 (92%)	Sugars, glycerol	Glycogen synthesis	Rnf, Sud, HppA	NiFe hydrogenase (Group 3d)
	meridiana	3300035698_985 (92%)				
Zapsychrus	exili^ T^	3300035698_1555 (89%)	Sugars, glycerol	Glycogen synthesis	Rnf, Nqr, Sud, HppA	NiFe hydrogenase (Group 3d)
Gygaella	obscura^ T^	3300035698_1934 (85%)	Sugars, 2-oxoacids	Glycogen synthesis	Rnf, Sud, HppA	FeFe hydrogenase (Group A3)
Susulua	stagnicola^ T^	3300035698_1005 (82%)	Sugars, 2-oxoacids	Glycogen synthesis	Rnf, Sud, HppA	FeFe hydrogenase (Group A3)
Saelkia	tenebricola^ T^	3300035698_749 (91%)	Sugars, alcohols	Glycogen synthesis	Rnf, Mrp, Sud, HppA	NiFe hydrogenase (Group 4g), Mrp-linked
Kaelpia	aquatica^ T^	3300035698_2000 (93%)	Sugars, 2-oxoacids, alcohols	Glycogen synthesis	Rnf, Sud, HppA	FeFe hydrogenase (Group A3)
	imicola	3300035698_1655 (92%)				
Kappaea	frigidicola^ T^	3300035698_1500 (76%)	Sugars	Trehalose synthesis	Rnf, HppA	FeFe hydrogenase (Group A3)
Tantalella	remota^ T^	3300035698_1097 (93%)	Sugars, glycerol, 2-oxoacids, alcohols	Glycogen synthesis, trehalose synthesis	Rnf, Sud, HppA	NiFe hydrogenase (Group 3b)/sulfhydrogenase
Aadella	gelida^ T^	3300035698_848 (91%)	Sugars, glycerol, 2-oxoacids	Trehalose synthesis	Rnf, Sud	NiFe hydrogenase (Group 3b)/sulfhydrogenase
Gorgyraea	atricola^ T^	3300035698_32 (93%)	Sugars, 2-oxoacids	Wood–Ljungdahl pathway, glycogen synthesis, trehalose synthesis	Rnf, Mrp, Sud, HppA	NiFe hydrogenase (Group 4g), Mrp-linked
Orphnella	occulta^ T^	3300025586_21 (85%), 3300035698_104 (61%)	Sugars, 2-oxoacids	Trehalose synthesis	Rnf, Sud, HppA	NiFe hydrogenase (Group 3b)/sulfhydrogenase

*HppA, pyrophosphate-energized sodium pump; Mrp, multicomponent Na^+^:H^+^ antiporter; Nqr, sodium-translocating NADH:quinone oxidoreductase; Rnf, ferredoxin:NAD^+^-oxidoreductase complex; Sud, bifunctional sulfide dehydrogenase/ferredoxin:NADP oxidoreductase. ^T^Indicates type species.*

For both Cloacimonadota and Omnitrophota, individual genera and species are provided along with etymologies of all proposed names (Tables 1, 2 and Supplementary Table S2). Proteins and pathways discussed for all MAGs assigned to each genus are provided for Cloacimonadota (Supplementary Tables S3, S4) and Omnitrophota (Supplementary Tables S5, S6).

### Cloacimonadota: Biopolymer Degradation and the Cell Envelope

Cloacimonadota are here inferred to be capable of degrading recalcitrant organic matter under anoxic conditions in Ace Lake. The MAGs of the four Ace Lake genera encode multiple glycoside hydrolase (GH) enzymes with signal peptides (indicating an extracytoplasmic location) and include enzymes that degrade polysaccharides and certain glycoconjugates, which would release oligosaccharides and simple sugars such as glucose (Table 1). These hydrolytic enzymes belong to various GH families, indicating a range of potential substrates, such as starch, β-glucans, β-glucosides, chitin, and glucosylceramides (Table 1 and Supplementary Tables S3, S4). The Ace Lake Cloacimonadota also encode diverse proteases and peptidases, including both secreted and cytoplasmic, indicating that polypeptides could be used as amino acid sources (Supplementary Tables S3, S4). Histidine degradation pathways are encoded in MAGs of all four genera, and *Ca.* Celaenobacter *gen. nov.* encodes proteins for tryptophan degradation (Supplementary Table S3). The abilities of Cloacimonadota to use complex sugars and proteins as organic substrates have been previously reported for this clade in both anaerobic digestors and lakes (Pelletier et al., 2008; Limam et al., 2014; Suominen et al., 2021).

**FIGURE 3:**
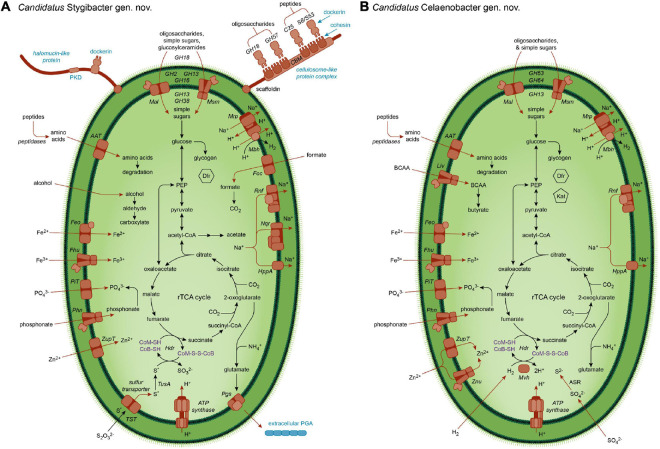
Metabolic capacity of Cloacimonadota. Metabolic capacities inferred from MAGs for **(A)** *Candidatus* Stygibacter and **(B)** *Candidatus* Celaenobacter. AAT, amino acid transporter; ABC, ATP-binding cassette transport system; BCAA, branched-chain amino acids; CoB-SH, coenzyme B; CoM-SH, coenzyme M; CoM-S-S-CoB, heterodisulfide; Dfr, desulfoferrodoxin; Feo, ferrous iron transporter; Fhu iron ABC transporter; Foc, formate transporter; GH, glycoside hydrolase; Hdr, heterodisulfide reductase; HppA, pyrophosphate-energized sodium pump; Mal, maltooligosaccharide transport system (permease + solute-binding protein); Kat, catalase; Liv, branched-chain amino acid ABC transporter; Mbh, membrane-bound [NiFe] hydrogenase; Mrp, multicomponent Na^+^:H^+^ antiporter; Msm, multiple sugar ABC transporter; Mvh, cytoplasmic [NiFe] hydrogenase; Nqr, sodium-translocating NADH:quinone oxidoreductase; PEP, phosphoenolpyruvate; PGA, poly-γ-glutamate; Pgs, poly-γ-glutamate synthase; Phn, phosphonate ABC transporter; PiT, inorganic phosphate transporter; PKD, Polycystic Kidney Disease domain (implicated in adhesion); Rnf, ferredoxin:NAD^+^-oxidoreductase complex; rTCA cycle, reverse tricarboxylic acid cycle; S*, unknown sulfur species; TST, thiosulfate:sulfurtransferase (rhodanese-like) (periplasmic); Znu, zinc ABC transporter; ZupT, zinc transporter. Note that the precise sulfur oxidation pathway for **(B)** is speculative; thiosulfate is shown here as the source of reductant, but the identity of the sulfur species is unclear.

However, in MAGs of the Ace Lake genus *Ca.* Stygibacter *gen. nov.* we identified genes for components of a putative extracellular, multienzyme complex for the binding and degradation of biopolymers (Table 1 and Figure 3A), not previously reported for Cloacimonadota. In *Ca.* Stygibacter, certain signal-peptide-bearing enzymes contain C-terminal dockerin domains: chitinase homolog (GH18), α-amylase/α-mannosidase homolog (GH57), serine peptidase (Peptidase S8/S53 domain), and gingipain-like peptidase (Peptidase C25). *Ca.* Stygibacter also encodes a large (3,755 amino acids) non-catalytic scaffoldin-like protein (Artzi et al., 2017) that contains tandemly repeated cohesin and carbohydrate-binding (CBM2/CBM3) domains. We infer that these dockerin- and cohesin-domain proteins combine to produce a cellulosome-like structure, with biopolymer-degrading enzymes integrated into this scaffoldin-like protein via complementary cohesin–dockerin interactions (Artzi et al., 2017).

Canonical cellulosomes are extracellular complexes equipped with cellulose-, hemicellulose-, and polypeptide-targeting enzymes that are used by cellulolytic Clostridia (Firmicutes) to bind and degrade plant cell walls (Schwarz and Zverlov, 2006; Peer et al., 2009; Fontes and Gilbert, 2010). As in clostridial cellulosomes, the *Ca.* Stygibacter complex has a scaffoldin-like protein with tandem cohesin domains for integration of multiple dockerin-containing enzymes, and a conserved C-terminal domain (CTD) for direct attachment to its own cell surface (Lasica et al., 2017). In common with the clostridial cellulosome, we infer that the cellulosome-like structure in *Ca.* Stygibacter can mediate attachment of cells to insoluble substrates and promote degradation to soluble products that are taken up by the cell (Lamed et al., 1983). Unlike cellulosomes, we propose that, based on the annotated GHs and peptidases, this complex in *Ca.* Stygibacter is utilized for the attachment to and degradation of starch (via endohydrolysis) and chitinous and proteinaceous material rather than cellulose-rich plant-derived material. A non-canonical cellulosome-like structure (“planctosome”) has also been reported in certain freshwater Planctomycetes (Nemodlikiaceae) for polypeptide degradation (Andrei et al., 2019), also distinct from the *Ca.* Stygibacter structure described here; our finding for the Ace Lake *Ca.* Stygibacter therefore adds to the repertoire of cellulosome-like complexes represented across the domain Bacteria.

The Ace Lake Cloacimonadota MAGs also encode other putative extracellular structures not previously reported for this phylum, all of which indicate an elaborate cell envelope (Figure 3A). Three genera (*Ca.* Stygibacter, *Ca.* Tenebribacter *gen. nov*., *Ca.* Zophobacter *gen. nov*.) encode poly-γ-glutamate synthetase (CapBC) and other proteins required for synthesis and transport of poly-γ-glutamate (PGA), a biopolymer involved in capsule formation or released extracellularly as a water-binding component of a biofilm matrix (Rehm, 2010). The water-binding properties of PGA allow it to locally decrease high salt concentrations, allowing survival of the cell in high-salt environments (Kandler et al., 1983; Rehm, 2010). Additionally, the *Ca.* Stygibacter MAGs encode a glycine-rich protein (944 amino acids), with the N-terminal half containing a ∼440 amino acid region that has 27–30% identity to a ∼400–500 amino acid repeat sequence in halomucin (a very large protein in the halophilic archaeon *Haloquadratum walsbyi*) (Bolhuis et al., 2006), and the C-terminal half includes a dockerin domain. As proposed for *H. walsbyi*, it is possible that both PGA and the halomucin-like protein form a water-enriched capsule around the cell that facilitates growth in high concentrations of salt (Bolhuis et al., 2006). These cell envelope features combined with Na^+^-translocating homeostasis abilities (see section “Cloacimonadota: Fermentation”) may assist growth of Cloacimonadota at the bottom of the Ace Lake water column where salinity is at its highest (43 g L^–1^) (Rankin et al., 1999; Lauro et al., 2011).

### Cloacimonadota: Fermentation

A fermentative metabolism is inferred for the Ace Lake Cloacimonadota, by which energy is derived from the oxidation of organic substrates (sugars, amino acids, 2-oxoacids, aldehydes, alcohols, and formate), and protons are used as the electron acceptor (Calusinska et al., 2010; Table 1 and Figures 3A,B). Simple sugars and amino acids, including those generated by extracellular degradation of biopolymers, appear to be principally imported by secondary transport, although a complete primary transporter for branched-chain amino acids (BCAAs) was identified in *Ca.* Celaenobacter MAGs. For simple sugars, the Embden–Meyerhof–Parnas (EMP) pathway for glycolysis generates NADH and reduced ferredoxin, as well as anabolic precursors (such as for the pentose phosphate pathway). There is no evidence in any of the 10 Ace Lake Cloacimonadota MAGs of a capacity for propionate oxidation, unlike members of *Ca.* Cloacimonadaceae (Pelletier et al., 2008; Dyksma and Gallert, 2019).

*Ca.* Tenebribacter, *Ca.* Stygibacter, and *Ca.* Zophobacter MAGs encode a membrane-bound [NiFe] hydrogenase (Mbh) (Group 4g) (Søndergaard et al., 2016). It has been proposed that Mbh transfers electrons from reduced ferredoxin to protons, thereby producing H_2_ gas; this would generate a Na^+^ gradient across the cell membrane via a Mrp-type Na^+^/H^+^ antiporter module (Mayer and Müller, 2014; Søndergaard et al., 2016; Yu et al., 2018). All four genera encode the Rnf complex, which couples electron transfer from reduced ferredoxin to NAD^+^ to generate NADH, with concomitant translocation of Na^+^ ions across the membrane (Biegel et al., 2011). The Na^+^ gradient drives ATP synthesis via a Na^+^-dependent F-type ATP synthase (Meier et al., 2009). This Na^+^ gradient can also be used for other purposes, such as phosphate uptake via a Na^+^/phosphate cotransporter (NptA-like). NADH can also be used for anabolic purposes. Additionally, *Ca.* Tenebribacter, *Ca.* Stygibacter, and *Ca.* Zophobacter encode a Na^+^-translocating NADH:quinone oxidoreductase complex (NQR), which couples NADH re-oxidation to Na^+^-extrusion, as well as maintaining ionic balance inside the cell (Verkhovsky and Bogachev, 2010).

All 10 Ace Lake Cloacimonadota MAGs lack genes for two essential enzymes of the oxidative tricarboxylic acid (TCA) cycle: citrate synthase and succinate dehydrogenase. Thus, we infer that they cannot operate a complete oxidative TCA cycle. All four genera encode phosphoenolpyruvate (PEP) carboxykinase, which converts oxaloacetate to PEP. The Ace Lake Cloacimonadota MAGs encode a pyrophosphate-dependent phosphofructokinase (PP_i_-PFK) as well as the more widely distributed ATP-dependent 6-phosphofructokinase (ATP-PFK), the former of which can reversibly function in both glycolysis and gluconeogenesis (Mertens, 1991; Kemp and Tripathi, 1993). Because PP_i_ is a byproduct of biosynthetic reactions, the use of PPi-PFK rather than ATP-PFK increases the energetic efficiency of glycolysis, especially during fermentation (Mertens, 1991; Reshetnikov et al., 2008). The presence of the reversible enzyme PP_i_-PFK is consistent with the absence of the gluconeogenesis-specific enzyme fructose-1,6-bisphosphatase from the Ace Lake Cloacimonadota MAGs. Having dual enzymes for the conversion of fructose-6-phosphate to fructose 1,6-bisphosphate might allow the Ace Lake Cloacimonadota to respond to the flux of high-energy phosphoryl donors in the cell (ATP vs. PP_i_). Furthermore, PP_i_ may also be diverted directly to energy conservation using a PP_i_-dependent Na^+^ pump (HppA) that utilizes the energy of PP_i_ hydrolysis as the driving force for Na^+^ translocation.

In addition to the abilities to ferment sugars, the Ace Lake Cloacimonadota MAGs encode multiple ferredoxin oxidoreductases that oxidize 2-oxoacids (including products of amino acid degradation), as inferred for *Ca.* Cloacimonas (Pelletier et al., 2008). These include pyruvate:ferredoxin oxidoreductase (POR); 2-oxoglutarate:ferredoxin oxidoreductase (OGOR); branched-chain 2-oxoacid (2-oxoisovalerate):ferredoxin oxidoreductase (VOR); indolepyruvate:ferredoxin oxidoreductase; and aldehyde:ferredoxin oxidoreductase (Supplementary Tables S3, S4). In addition to VOR, the Ace Lake Cloacimonadota encode phosphate butyryltransferase and butyrate kinase, suggesting the potential for further catabolism of branched-chain 2-oxoacids derived from degradation of BCAAs.

*Ca.* Stygibacter and *Ca.* Zophobacter MAGs encode phosphate acetyltransferase and acetate kinase for the conversion of acetyl-CoA to acetate *via* acetyl phosphate with concomitant production of ATP *via* substrate-level phosphorylation (Sapra et al., 2003), also inferred for Cloacimonadota MAG TCS47 (Zhang et al., 2016). In addition to Mbh, two reversible [FeFe] cytoplasmic hydrogenases were identified in certain Ace Lake Cloacimonadota: a tetrameric Group A3 hydrogenase (*Ca.* Stygibacter) and a monomeric Group C1 hydrogenase (*Ca.* Tenebribacter and *Ca.* Stygibacter) (Søndergaard et al., 2016). As a bidirectional hydrogenase, the Group A3 hydrogenase could use H_2_ as an energy source through the bifurcation of electrons from H_2_ to ferredoxin and NAD^+^ (Poudel et al., 2016; Søndergaard et al., 2016; Kpebe et al., 2018), or it could serve as a confurcating hydrogenase to dissipate surplus reductant (from both NADH and reduced ferredoxin) that is generated during fermentation (Schut and Adams, 2009; Poudel et al., 2016). For the latter, substrate-level phosphorylation in the conversion of glucose to acetate would be facilitated by the dissipation of both reducing equivalents (NADH and reduced ferredoxin) as H_2_ (Herrmann et al., 2008). These findings indicate that the Ace Lake Cloacimonadota would generate H_2_ and acetate as byproducts of carbohydrate fermentation.

In both the *Ca.* Tenebribacter and *Ca.* Stygibacter MAGs, the Group C1 [FeFe] hydrogenase gene is immediately downstream of a gene for a histidine kinase domain protein, providing support for a putative sensory function (Greening et al., 2016). However, in one *Ca.* Tenebribacter MAG (3300035698_1346) the same gene cluster also encodes homologs of hydrogenase subunits associated with electron bifurcation (Poudel et al., 2016), which raises the possibility of a metabolic role for the Group C1 [FeFe] hydrogenase.

### Cloacimonadota: Carbon Fixation Using a Reverse Tricarboxylic Acid Cycle

The gene inventories of certain Ace Lake Cloacimonadota suggest that they are capable of operating the reverse tricarboxylic acid (rTCA) cycle for carbon fixation, driven by sulfur oxidation (*Ca.* Tenebribacter and *Ca.* Stygibacter) (Figure 3A) or H_2_ oxidation (*Ca.* Celaenobacter) (Figure 3B). The MAGs of these three genera encode ATP citrate lyase (ACL), thiol:fumarate reductase (TFR), and OGOR; these three enzymes allow the TCA cycle to proceed in the reductive direction (Rubin-Blum et al., 2019). PEP carboxykinase would connect the rTCA cycle to gluconeogenesis (Marietou et al., 2020) (see section “Cloacimonadota: Fermentation”). In the *Ca.* Celaenobacter MAGs, the genes for TFR and OGOR are part of a gene cluster that also includes fumarate hydratase, succinyl-CoA synthetase, [NiFe] hydrogenase (Mvh) (Group 3c), and heterodisulfide reductase (Hdr), which is consistent with a functional link between all these proteins. ACL is encoded elsewhere in the *Ca.* Celaenobacter genome, in a gene cluster that also includes the TCA cycle enzymes aconitase and isocitrate dehydrogenase. In general, the cytoplasmic Mvh hydrogenase forms a complex with Hdr, and bifurcates electrons from H_2_ to heterodisulfide (CoM-S-S-CoB) and ferredoxin; the Mvh–Hdr complex couples the exergonic reduction of heterodisulfide with the endergonic reduction of ferredoxin with H_2_ (Heim et al., 1998; Kaster et al., 2011; Greening et al., 2016). Thus, in *Ca.* Celaenobacter, carbon fixation using the rTCA cycle would be driven by H_2_ oxidation (Figure 3B), as in *Aquifex aeolicus* (Brugna-Guiral et al., 2003; Guiral et al., 2005), although the latter have been inferred to use a Group 2d cytoplasmic hydrogenase for carbon fixation (Greening et al., 2016). ACL, OGOR, TFR, and Hdr genes were also identified in MAGs assigned to *Ca.* Tenebribacter and *Ca.* Stygibacter. Hdr genes in one *Ca.* Stygibacter MAG (3300025642_13) are within a gene cluster that also contains genes implicated in sulfur metabolism, including thiosulfate:sulfurtransferase (TST) (with a predicted signal peptide), a cytoplasmic sulfur relay protein TusA, and a sulfur compound transporter (Gristwood et al., 2011; Tanaka et al., 2020). Homologs of these four proteins are also encoded in MAGs assigned to *Ca.* Tenebribacter. Thus, energy required for carbon fixation in *Ca.* Tenebribacter and *Ca.* Stygibacter may be derived from sulfur oxidation catalyzed by TST and Hdr, as in certain other autotrophic bacteria (Boughanemi et al., 2016; Koch and Dahl, 2018; Wang et al., 2019). Based on the presence of a TST homolog in these Ace Lake Cloacimonadota MAGs, the electron donor may be thiosulfate, with the initial reaction occurring in the periplasm (Figure 3A); however, elemental sulfur might also be utilized, as in *A. aeolicus* (Boughanemi et al., 2016).

### Omnitrophota Ecophysiology

Based on interrogation of 14 Ace Lake Omnitrophota MAGs, this candidate phylum possesses a heterotrophic and fermentative metabolism. None of the MAGs possess any genes necessary for motility or magnetotaxis. We infer the Ace Lake Omnitrophota to be heterotrophs that are capable of fermenting a narrow range of substrates for energy conservation. All MAGs encode proteases and peptidases (some with signal peptides) to degrade proteins to amino acids. However, there are very few enzymes encoded for the catabolism of amino acids, and there is no evidence in any of the 14 MAGs of genes required for amino acid fermentation, unlike *Clostridium* spp. (Herrmann et al., 2008; Perret et al., 2011). Thus, we posit that these Ace Lake Omnitrophota use amino acids derived from peptide hydrolysis as nitrogen sources (especially by deamination or transamination) or for protein synthesis.

The Ace Lake Omnitrophota MAGs encode ABC transporter systems for sugars (disaccharides and/or oligosaccharides) and a number of GHs (including β-glucosidases and sugar phosphorolytic enzymes) to break down di- and oligosaccharides into simpler sugars such as glucose and/or glucose-1-phosphate (Table 2 and Supplementary Tables S5, S6). Enzymes necessary for the initial depolymerization of polysaccharides are absent from all MAGs, which suggests that the Ace Lake Omnitrophota are dependent on other microorganisms for initial degradation of biopolymers, as inferred for the Black Sea Omnitrophota (Suominen et al., 2021). Simple sugars imported into the cell could also be utilized by the Ace Lake Omnitrophota for the synthesis of the compatible solute trehalose, or for the synthesis of glycogen for carbon and energy storage; enzymes for both processes were encoded across the Omnitrophota MAGs (Table 2 and Supplementary Table S5).

The Ace Lake Omnitrophota MAGs also encode the capacity for fermentation of glucose to acetyl-CoA *via* the EMP pathway. The Rnf complex is predicted to couple the reduction of NAD^+^ to the oxidation of reduced ferredoxin and translocation of protons across the cell membrane, allowing ATP generation via a V-type ATP synthase. The majority of MAGs also encode the enzymes phosphate acetyltransferase and acetate kinase for the subsequent conversion of acetyl-CoA to acetate with concomitant production of ATP via substrate-level phosphorylation (Sapra et al., 2003).

**FIGURE 4:**
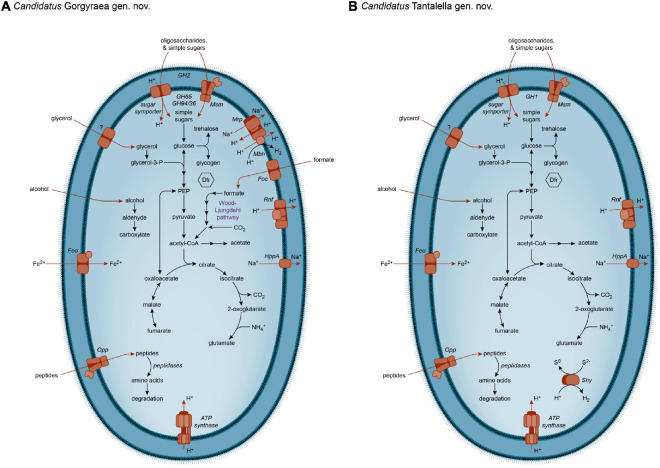
Metabolic capacity of Omnitrophota. Metabolic capacities inferred from MAGs for **(A)** *Candidatus* Gorgyraea and **(B)** *Candidatus* Tantalella. ABC, ATP-binding cassette transport system; Dfr, desulfoferrodoxin; Feo, ferrous iron transporter; Foc, formate transporter; GH, glycoside hydrolase; HppA, pyrophosphate-energized sodium pump; Mbh, membrane-bound [NiFe] hydrogenase; Mrp, multicomponent Na^+^:H^+^ antiporter; Msm, multiple sugar ABC transporter; Opp, oligopeptide ABC transporter; PEP, phosphoenolpyruvate; Rnf, ferredoxin:NAD^+^-oxidoreductase complex; Shy, sulfhydrogenase.

Excess reductant generated during fermentation could be dissipated as H_2_ using a cytoplasmic hydrogenase (Dombrowski et al., 2017; Suominen et al., 2021). The Ace Lake Omnitrophota MAGs encode various hydrogenases, although it is noteworthy that each MAG has only one identifiable hydrogenase: Group 3d [NiFe] hydrogenase (*Ca.* Aceula *gen. nov*. and *Ca.* Zapsychrus *gen. nov.*); Group 3b [NiFe] hydrogenase (*Ca*. Tantalella *gen. nov.*, *Ca.* Aadella *gen. nov.*, and *Ca.* Orphnella *gen. nov.*); Group 4g [NiFe] hydrogenase (*Ca.* Saelkia *gen. nov.*, and *Ca.* Gorgyraea *gen. nov.*), and Group A3 [FeFe] hydrogenase (*Ca.* Gygaella. *gen. nov*, *Ca.* Susulua *gen. nov.*, *Ca.* Kaelpia *gen. nov.*, and *Ca.* Kappaea *gen. nov.*). We infer that these hydrogenases are used for redox balance, associated with the need to dispose of surplus reductant. MAGs of both genera that encode Group 4g Mbh also encode Mrp (*Ca.* Saelkia and *Ca.* Gorgyraea) (Figure 4A); this hydrogenase may therefore function in a complex with the Mrp antiporter to generate an ionic gradient across the cell membrane, as in the other Mbh (Mayer and Müller, 2014; Søndergaard et al., 2016; Yu et al., 2018). The Group 3b hydrogenases of *Ca*. Tantalella, *Ca.* Aadella, and *Ca.* Orphnella were annotated as a bifunctional sulfhydrogenase (Shy) with dual hydrogenase and sulfur reductase activity (Figure 4B), meaning that excess reductant generated during fermentation can be disposed of as H_2_ and sulfide, respectively (Ma et al., 1993; Silva et al., 1999; Ma et al., 2000).

None of the 14 Ace Lake Omnitrophota MAGs encode a complete TCA cycle (Figures 4A,B), either in the oxidative or reverse directions, with OGOR, succinyl-CoA synthetase, succinate dehydrogenase, ACL, and fumarate reductase absent from all MAGs (Table 2). We infer that the Ace Lake Omnitrophota possess an incomplete, “horse-shoe”-type TCA cycle as found in certain other anaerobic bacteria (e.g., Herlemann et al., 2009; Marco-Urrea et al., 2011). The type of citrate synthase varies, with either (but never both) citrate (*Si*)-synthase or citrate (*Re*)-synthase (Li et al., 2007; Marco-Urrea et al., 2011) encoded in individual MAGs, with the distribution of the functional analogs mostly conforming to separate Omnitrophota clades (Supplementary Table S6). The right branch of the incomplete TCA pathway of Omnitrophota is inferred to occur in the oxidative direction and commence at citrate synthase and terminate at 2-oxoglutarate. The left branch allows the interconversion of oxaloacetate, malate, and fumarate (Herlemann et al., 2009). This could proceed in the oxidative direction, with fumarate (such as generated as a byproduct of arginine synthesis) converted to oxaloacetate and used for gluconeogenesis (van Vugt-Lussenburg et al., 2009). Alternatively, this left branch may operate in the reductive direction, and be initiated by PEP carboxykinase (Herlemann et al., 2009); the subsequent reduction of oxaloacetate to fumarate would provide redox balance to the oxidative branch (Meléndez-Hevia et al., 1996). The “horseshoe-type” TCA cycle has no energy conservation function but serves solely for biosynthesis (Wood et al., 2004). The carbon skeleton 2-oxoglutarate is required for ammonia assimilation, and all 14 MAGs encode enzymes for this process (Supplementary Tables S5, S6). However, the fate of fumarate in Omnitrophota is unclear; there is no identifiable fumarate reductase (for anaerobic respiration), fumarate-adding enzymes (for hydrocarbon degradation), or aspartase (for synthesis of aspartate directly from fumarate) in any of the 14 Omnitrophota MAGs.

Although autotrophic pathways have been inferred in other Omnitrophota (Rinke et al., 2013; Kolinko et al., 2016) (see section “Cloacimonadota and Omnitrophota in the Ace Lake Ecosystem”), the Ace Lake Omnitrophota appear to be obligate heterotrophs. *Ca.* Gorgyraea encodes WL pathway genes (Table 2, Figure 4A, and Supplementary Table S5), but in the absence of rTCA cycle genes that link acetyl-CoA to central biosynthetic pathways (Youssef et al., 2019), we infer that the WL pathway does not function in autotrophic CO_2_ fixation. Instead, we propose that the WL pathway, in combination with the Rnf complex, functions in the reductive direction as an electron sink during homoacetogenic glucose fermentation, to maintain redox balance (Schuchmann and Müller, 2016; Youssef et al., 2019).

Overall, we infer only minor differences in the metabolic strategies among the 14 Ace Lake Omnitrophota MAGs (e.g., ability to catabolize glycerol, alcohol, or certain sugars; mechanisms for redox balance) (Table 2). Thus, all 11 Omnitrophota genera described here appear to conform to the same metabolic template: fermentative heterotrophs capable of degradation of a narrow range of organic compounds (especially simple sugars), with a hydrogenase for redox balance. The biosynthetic potential of the Ace Lake Omnitrophota MAGs are impressive, with the genomic capacity to synthesize nucleosides, fatty acids, the majority of proteinogenic amino acids, and essential cofactors (Supplementary Table S5).

### Cloacimonadota and Omnitrophota in the Ace Lake Ecosystem

Although the ecophysiology of Ace Lake Cloacimonadota broadly agree with the fermentative, heterotrophic generalists inferred for members of this phylum from the Black Sea (Suominen et al., 2021), we infer a number of traits in certain Ace Lake Cloacimonadota that have not been previously reported for this candidate phylum. These include the presence of an extracellular cellulosome-like structure for the binding and degradation of biopolymers, PGA synthesis, a halomucin-like protein, and a chemolithoautotrophic pathway for carbon fixation *via* the rTCA cycle, fueled by oxidation of H_2_ or sulfur compounds. These abilities attest to the physical and metabolic diversity of the Cloacimonadota, and emphasize the potential importance of this group in cycling of carbon, hydrogen, and sulfur in Ace Lake.

**FIGURE 5:**
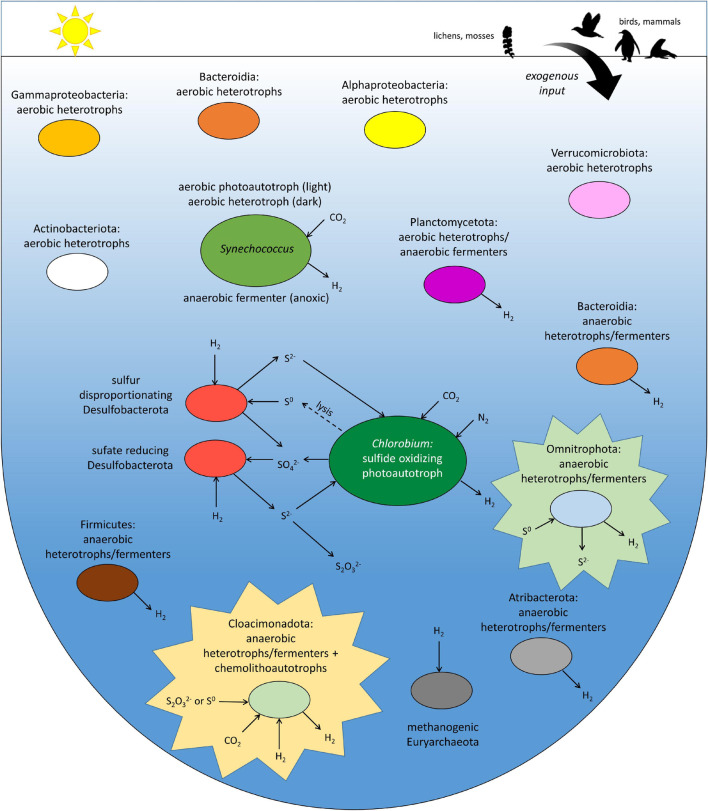
Depiction of the most abundant bacteria and archaea in Ace Lake in the austral summer. Sulfur and hydrogen cycles are emphasized. Sizes of the ellipses that represent individual clades are approximately proportional to their peak relative abundance in the lake (Panwar et al., 2020), with *Chlorobium* (Chlorobia) the most abundant and *Synechococcus* (Cyanobacteria) the second most abundant. *Chlorobium* marks the oxic–anoxic interface of the lake. The candidate phyla Cloacimonadota and Omnitrophota are highlighted. Different taxa within candidate phylum Cloacimonadota are inferred to be capable of hydrogen and sulfur oxidation for chemolithoautotrophic growth; hydrogen is produced during heterotrophic growth. All taxa within the Omnitrophota candidate phylum are inferred to generate hydrogen, but only some are inferred to generate sulfide. Animal and plant silhouettes are courtesy of PhyloPic (http://phylopic.org/).

*Chlorobium*, which is the dominant organism in Ace Lake, also employs the rTCA cycle; this anaerobic photoautotroph grows at the limits of the penetration of photosynthetically active radiation at the oxic–anoxic interface (Panwar et al., 2020; Figure 5). As a consequence, the abundance of *Chlorobium* in this lake system is dictated by the polar light cycle, with a peak relative abundance of 83% at the interface in summer, and a marked decline in winter (6%) to spring (1%) (Panwar et al., 2020). *Chlorobium* is not metabolically active in the perennially dark anoxic zone of Ace Lake, and sinks to the bottom as particulate matter (Rankin et al., 1999; Lauro et al., 2011; Panwar et al., 2020). By contrast, light-independent, facultative chemolithoautotrophs, including the Ace Lake Cloacimonadota, would not be directly impacted by the marked seasonal variation in light availability. Although Cloacimonadota were most abundant in the deepest part of the anoxic zone of Ace Lake, they were detected throughout the anoxic zone, as well as at the interface (Panwar et al., 2020). For those Cloacimonadota that we infer to use H_2_ oxidation for carbon fixation (*Ca.* Celaenobacter), *Chlorobium* is potentially a major source of H_2_, as a byproduct of nitrogen fixation by a membrane-bound nitrogenase (Lauro et al., 2011; Panwar et al., 2020). There are other bacteria present throughout the anoxic zone of Ace Lake that have been inferred to be H_2_-evolving obligate anaerobes; aside from Omnitrophota, these include members of Bacteroidia, Firmicutes, and Atribacterota (Panwar et al., 2020). The cyanobacterium *Synechococcus*, the most abundant phototrophic bacterium in the oxic zone of Ace Lake, was also abundant in the interface and anoxic zone, and previously inferred to be capable of fermentation coupled to H_2_ production (Panwar et al., 2020); thus, *Synechococcus* is also a potential source of H_2_ for H_2_-oxidizing Cloacimonadota. For those Ace Lake Cloacimonadota that we infer to rely on sulfur oxidation for carbon fixation (*Ca.* Tenebribacter and *Ca.* Stygibacter), *Chlorobium* cells could be a source of elemental sulfur and polysulfide, as intermediates in sulfide oxidation (Marnocha et al., 2016), whereas thiosulfate is likely generated from the chemical reaction of sulfide (such as those generated by sulfate-reducing Desulfobacterota) with dissolved oxygen (Kondo et al., 2000). However, unlike sulfate-reducing and sulfur-disproportionating Desulfobacterota in Ace Lake, which were previously inferred to be metabolically linked to *Chlorobium* via sulfur cycling (Lauro et al., 2011; Panwar et al., 2020; Figure 5), no seasonal variation was observed for Cloacimonadota in Ace Lake. Although Cloacimonadota and Omnitrophota in Ace Lake are inferred to be obligate anaerobes, both are found in the oxic–anoxic interface; the MAGs encode mechanisms to help protect against oxidative stress (e.g., desulfoferrodoxin in both phyla, catalase in Cloacimonadota), allowing survival at minimal oxygen concentrations (Pelletier et al., 2008). Thus, we infer that these anaerobes are aerotolerant, as previously inferred for *Chlorobium* in Ace Lake (Ng et al., 2010).

During heterotrophic growth, the anaerobic degradation of hexoses to acetate, CO_2_, and H_2_ does not yield sufficient energy to support growth unless H_2_ levels are sufficiently low (Thauer et al., 1977; Schink, 1997; Morris et al., 2013). Acetate and H_2_ would be beneficial to hydrogenotrophic and acetoclastic methanogens (Euryarchaeota), which, like Cloacimonadota, are most abundant in the deepest waters of Ace Lake (Panwar et al., 2020; Figure 5); as such, we posit that Cloacimonadota and methanogens may be metabolically linked. The extracellular GHs and peptidases, including the extracellular cellulosome-like structure inferred for the Cloacimonadota genus *Ca.* Stygibacter, suggest that Cloacimonadota are among the “first responders” in deconstructing and assimilating recalcitrant particulate organic matter, including microbial aggregates that sink to the bottom from higher in the water column (Rankin et al., 1999; Lauro et al., 2011).

All the Ace Lake Omnitrophota MAGs appear to be obligate heterotrophs, with no evidence of autotrophic capacity. Furthermore, these anaerobes appear to be dependent on hydrogenotrophic microbes to consume H_2_ released *via* anaerobic glucose fermentation. In Ace Lake, Omnitrophota were most abundant at the oxic–anoxic interface and the water column immediately below the interface, the depths at which sulfate-reducing and sulfur-disproportionating Desulfobacterota were also most abundant (Panwar et al., 2020; Figure 5). These Desulfobacterota encode H_2_-uptake hydrogenases (Panwar et al., 2020), and would therefore be capable of consuming H_2_.

The ability to infer the ecophysiology of the members of Cloacimonadota and Omnitrophota emphasizes the importance of having metagenome data and accompanying analyses detailing the metabolisms of numerous other members of the microbial community (Ng et al., 2010; Lauro et al., 2011; Panwar et al., 2020). Here, we added to the understanding of the system by analyzing a total of 24 MAGs for these “microbial dark matter” lineages. Both of these candidate phyla are predicted to engage in metabolic associations with other Ace Lake microorganisms. The specific involvement of hydrogen is noteworthy in view of hydrogen cycling being previously identified as pivotal to multiple nutrient cycles in Ace Lake (Panwar et al., 2020), as well as being increasingly recognized as important to ecosystem function in global anoxic and oxic environments (Greening et al., 2016).

## Data Availability Statement

The datasets presented in this study can be found in online public repositories. The repository and accession numbers are: IMG (https://img.jgi.doe.gov/) (Metagenomes 3300035698, 3300025586, 3300025698, and 3300025642; MAGs 330 0035698_1174, 3300035698_1346, 3300035698_1468, 3300035 698_198, 3300035698_2003, 3300025642_13, 3300035698_360, 3300035698_1703, 3300035698_1683, 3300025698_8, 33000356 98_1100, 3300035698_985, 3300035698_1555, 3300035698_19 34, 3300035698_1005, 3300035698_749, 3300035698_2000, 3300035698_1655, 3300035698_1500, 3300035698_1097, 3300 035698_848, 3300035698_32, 3300025586_21, and 3300035 698_104).

## Author Contributions

TW, MA, and RC conceived the study, analyzed the data, and/or conducted the data interpretation. JB provided illustrations. TW, MA, and RC wrote the manuscript with input from all authors. All authors have read and approved the manuscript submission.

## Conflict of Interest

The authors declare that the research was conducted in the absence of any commercial or financial relationships that could be construed as a potential conflict of interest.

## Publisher’s Note

All claims expressed in this article are solely those of the authors and do not necessarily represent those of their affiliated organizations, or those of the publisher, the editors and the reviewers. Any product that may be evaluated in this article, or claim that may be made by its manufacturer, is not guaranteed or endorsed by the publisher.

## Footnotes

1
https://github.com/dparks1134/CompareM
