# Supplementary material for: Shedding Light on Microbial “Dark Matter”: Insights Into Novel Cloacimonadota and Omnitrophota From an Antarctic Lake
Source: Front Microbiol. 2021 Oct 11;12:741077. doi: 10.3389/fmicb.2021.741077 (PMC8542988; doi:10.3389/fmicb.2021.741077)
Supplement: Supplementary file 5 [file Data_Sheet_1.PDF]

Supplementary Material

Shedding Light on Microbial 'Dark Matter': Insights into Novel Cloacimonadota and Omnitrophota from an Antarctic Lake

Timothy J. Williams, Michelle A. Allen, Jonathan F. Berengut and Ricardo Cavicchioli

1 Supplementary Figures

Figure S1 | Expanded phylogeny of phylum *Candidatus* Cloacimonadota.

Figure S2 | Expanded phylogeny of phylum *Candidatus* Omnitrophota.

2 Supplementary Tables

Table S1 | Description of all Ace Lake Cloacimonadota and Omnitrophota MAGs. (see accompanying excel file)

Table S2 | Etymologies and metadata for proposed *Candidatus* genus and species Cloacimonadota and Omnitrophota from Ace Lake. (see accompanying excel file)

Table S3 | Proteins and IMG Gene IDs for the ten Ace Lake Cloacimonadota MAGs chosen for analysis in this study. (see accompanying excel file)

Table S4 | Metabolic traits inferred from analysis of Ace Lake Cloacimonadota MAGs.

Table S5 | Proteins and IMG Gene IDs for the 14 Ace Lake Omnitrophota MAGs chosen for analysis in this study. (see accompanying excel file)

Table S6 | Metabolic traits inferred from analysis of Ace Lake Omnitrophota MAGs.

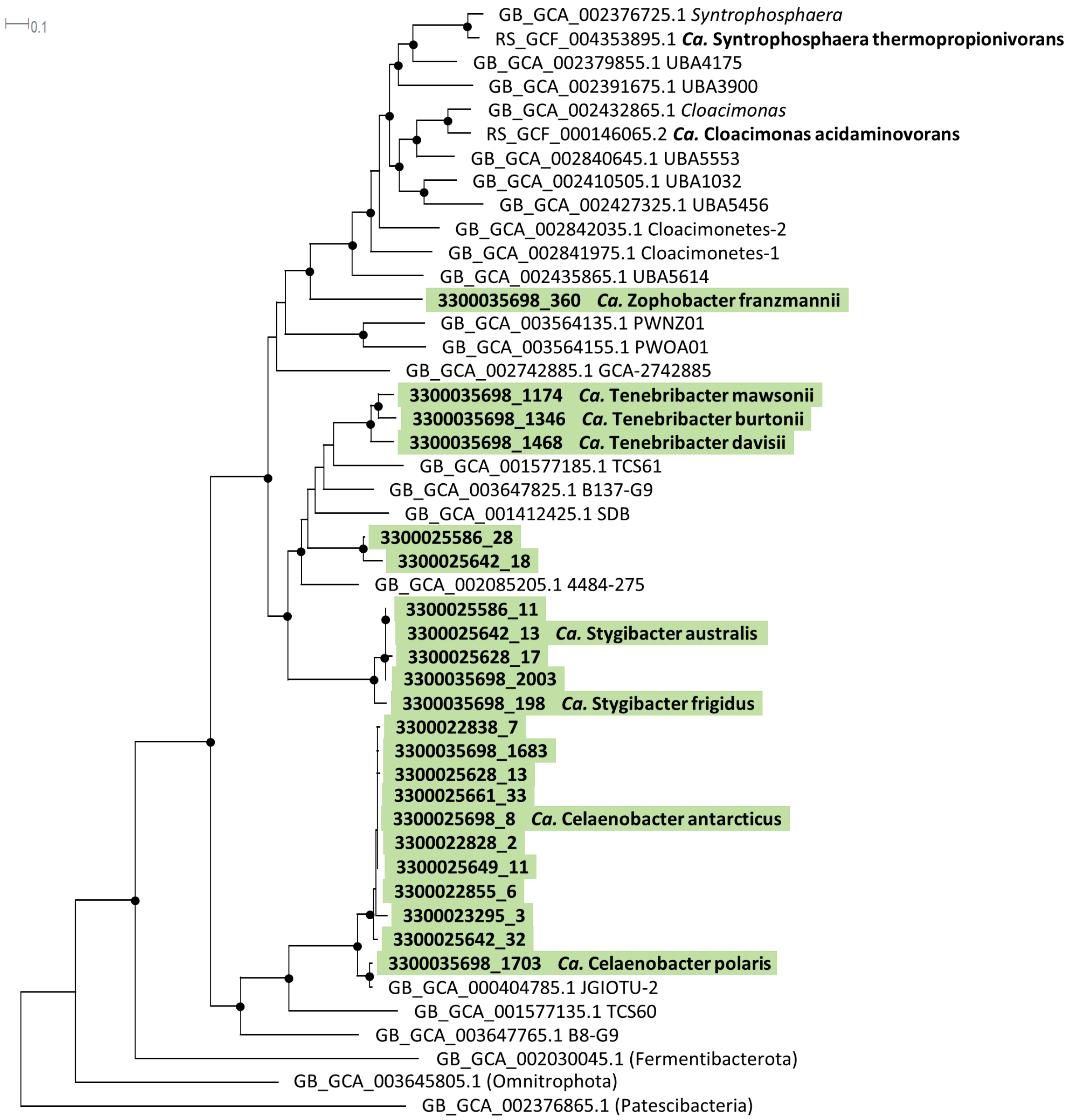

Figure S1 | Expanded phylogeny of phylum *Candidatus* Cloacimonadota. Maximum likelihood tree constructed in IQ-Tree with autoselection of the best-fit model (LG+F+I+G4) and 1000 ultra-fast bootstraps. UFBootstraps ≥ 95% (black dot); Metagenome-assembled genomes (MAGs) featured in this study (green) with their IMG MAG ID and proposed *Candidatus* genus and species name. Reference Cloacimonadota MAGs are shown with their Genome Taxonomy Database (GTDB) accession and GTDB taxonomy, except for *Candidatus* Cloacimonas acidaminovorans and *Candidatus* Syntrophosphaera thermopropionivorans, which already have names. The tree is rooted using a representative of the Patescibacteria.

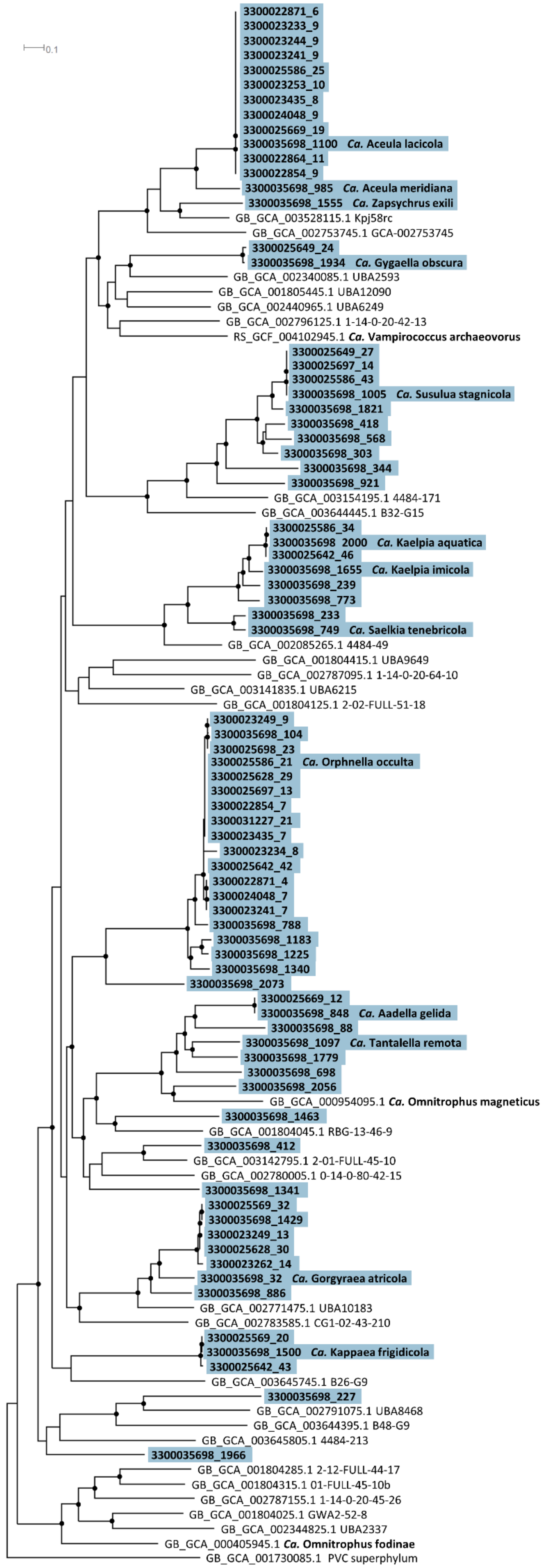
0.1
3300022871_6
3300023233_9
3300023244_9
3300023241_9
3300025586_25
3300023253_10
3300023435_8
3300024048_9
3300025669_19
3300035698_1100 Ca. Aceula lacicola
3300022864_11
3300022854_9
3300035698_985 Ca. Aceula meridiana
3300035698_1555 Ca. Zapsychrus exili
GB_GCA_003528115.1 Kpj58rc
GB_GCA_002753745.1 GCA-002753745
3300025649_24
3300035698_1934 Ca. Gygaella obscura
GB_GCA_002340085.1 UBA2593
GB_GCA_001805445.1 UBA12090
GB_GCA_002440965.1 UBA6249
GB_GCA_002796125.1 1-14-0-20-42-13
RS_GCF_004102945.1 Ca. Vampirococcus archaeovorus
3300025649_27
3300025697_14
3300025586_43
3300035698_1005 Ca. Susulua stagnicola
3300035698_1821
3300035698_418
3300035698_568
3300035698_303
3300035698_344
3300035698_921
GB_GCA_003154195.1 4484-171
GB_GCA_003644445.1 B32-G15
3300025586_34
3300035698_2000 Ca. Kaelpia aquatica
3300025642_46
3300035698_1655 Ca. Kaelpia imicola
3300035698_239
3300035698_773
3300035698_233
3300035698_749 Ca. Saelkia tenebricola
GB_GCA_002085265.1 4484-49
GB_GCA_001804415.1 UBA9649
GB_GCA_002787095.1 1-14-0-20-64-10
GB_GCA_003141835.1 UBA6215
GB_GCA_001804125.1 2-02-FULL-51-18
3300023249_9
3300035698_104
3300025698_23
3300025586_21 Ca. Orphnella occulta
3300025628_29
3300025697_13
3300022854_7
3300031227_21
3300023435_7
3300023234_8
3300025642_42
3300022871_4
3300024048_7
3300023241_7
3300035698_788
3300035698_1183
3300035698_1225
3300035698_1340
3300035698_2073
3300025669_12
3300035698_848 Ca. Aadella gelida
3300035698_88
3300035698_1097 Ca. Tantalella remota
3300035698_1779
3300035698_698
3300035698_2056
GB_GCA_000954095.1 Ca. Omnitrophus magneticus
3300035698_1463
GB_GCA_001804045.1 RBG-13-46-9
3300035698_412
GB_GCA_003142795.1 2-01-FULL-45-10
GB_GCA_002780005.1 0-14-0-80-42-15
3300035698_1341
3300025569_32
3300035698_1429
3300023249_13
3300025628_30
3300023262_14
3300035698_32 Ca. Gorgyraea atricola
3300035698_886
GB_GCA_002771475.1 UBA10183
GB_GCA_002783585.1 CG1-02-43-210
3300025569_20
3300035698_1500 Ca. Kappaea frigidicola
3300025642_43
GB_GCA_003645745.1 B26-G9
3300035698_227
GB_GCA_002791075.1 UBA8468
GB_GCA_003644395.1 B48-G9
GB_GCA_003645805.1 4484-213
3300035698_1966
GB_GCA_001804285.1 2-12-FULL-44-17
GB_GCA_001804315.1 01-FULL-45-10b
GB_GCA_002787155.1 1-14-0-20-45-26
GB_GCA_001804025.1 GWA2-52-8
GB_GCA_002344825.1 UBA2337
GB_GCA_000405945.1 Ca. Omnitrophus fodinae
GB_GCA_001730085.1 PVC superphylum

Figure S2 | Expanded phylogeny of phylum *Candidatus* Omnitrophota. Maximum likelihood tree constructed in IQ-Tree with autoselection of the best-fit model (LG+F+I+G4) and 1000 ultra-fast bootstraps. UFBootstraps $\geq$ 95% (black dot); Metagenome-assembled genomes (MAGs) featured in this study (blue) with their IMG MAG ID and proposed *Candidatus* genus and species names. Reference Omnitrophota MAGs are shown with their Genome Taxonomy Database (GTDB) accession and GTDB taxonomy, except for *Candidatus* Omnitrophus fodinae and *Candidatus* Omnitrophus magneticus, which already have names. The tree was rooted with a basal representative of the 'Planctomycetes-Verrucomicrobia-Chlamydiae' (PVC) superphylum.

Table S4 | Metabolic traits inferred from analysis of Ace Lake Cloacimonadota MAGs.

Class (GTDB)	Cloacimonadia									
Order (GTDB)	Cloacimonadales						JGIOTU-2			
Family (GTDB)	TCS61			--			JGIOTU-2			
Candidatus genus	Tenebribacter			Stygibacter			Zophobacter	Celaenobacter		
Candidatus species	burtonii (type)	mawsonii	davisii	australis (type)		frigidus	franzmannii (type)	antarcticus (type)		polaris
MAG ID	3300035698_1346 (type)	3300035698_1174 (type)	3300035698_1468 (type)	3300025642_13 (type)	3300035698_2003	3300035698_198 (type)	3300035698_360 (type)	3300025698_8 (type)	3300035698_1683	3300035698_1703 (type)
Genome completeness	97%	91%	97%	88%	69%	58%	80%	92%	65%	91%
Genome contamination	0%	0%	1.7%	3.3%	2.8%	0%	1.3%	4.4%	1.3%	1.1%
Cell surface	PGA	PGA	PGA	PGA, cellulosome-like, halomucin-like*	PGA, cellulosome-like, halomucin-like*	PGA, cellulosome-like	PGA			
rTCA cycle	ACL	TFR		TFR	TFR	ACL, TFR		ACL, TFR	ACL	TFR
Hydrogenases	FeFe Group C1	NiFe Group 4g (Mbh)	FeFe Group C1	NiFe Group 4g (Mbh), FeFe Group A3 (Hnd), FeFe Group C1	NiFe Group 4g (Mbh), FeFe Group A3 (Hnd), FeFe Group C1	FeFe Group A3 (Hnd)		NiFe Group 4g (Mbh), NiFe Group 3c (Mvh)		NiFe Group 4g (Mbh), NiFe Group 3c (Mvh)
Sulfur metabolism	TST	Hdr, TST, TusA	TST	Hdr, TST, TusA	Hdr, TST, TusA	Hdr, TusA		Hdr, Sat+CysC	Hdr, Sat+CysC	Hdr, CysC
Ferredoxin oxidoreductases	POR, OGOR, IOR, AOR	POR, OGOR, VOR, IOR, AOR	POR, VOR, IOR, AOR	POR, OGOR, VOR, IOR, AOR	POR, OGOR, IOR, AOR	OGOR, VOR, IOR	OGOR, VOR, IOR	POR, OGOR, IOR	POR, OGOR, IOR	POR, OGOR, IOR
SCFA catabolism	Ptb+Buk, Fdh	Ptb+Buk	Ptb+Buk, Fdh	Ptb+Buk, Pta+Ack, Fdh	Ptb+Buk, Pta+Ack, Fdh	Ptb, Fdh	Ptb+Buk, Pta+Ack,	Ptb+Buk	Ptb	Ptb+Buk
Energy conservation	Rnf, Mrp, HppA, Nqr, Sud	Rnf, HppA, Nqr, Sud	Rnf, HppA, Nqr, Sud	Rnf, HppA, Nqr,	Rnf, Mrp, HppA, Nqr, Sud	Mrp, HppA, Nqr, Sud	Rnf, HppA, Nqr, Sud	Rnf, Mrp, HppA,	Rnf, Mrp	Rnf, Mrp, Sud
Glycerol catabolism	GlpK	GlpK	GlpK	GlpK			GlpK			
Alcohol catabolism	Adh			Adh	Adh	Adh				
Poly/ oligosaccharide degradation (glycoside hydrolases)	GH18, GH38	GH13	GH16, GH3, GH30, GH38	GH16, GH2, GH13, GH3, GH30, GH31, GH38 GH57*, GH18*	GH16, GH2, GH18, GH13, GH38, GH57*, GH18*	GH16, GH2, GH13, GH3, GH30, GH57*, GH18*	GH16, GH18, GH3, GH27, GH30, GH31, GH92	GH64, GH53	GH64	GH13
Polypeptide degradation (proteases/ peptidases)	S1, S9A, S49, M16, M18, M20, M24, M42	S1, S9A, S49, M16, M18, M20, M24, M42	S1, S9A, S49, M16, M18, M20, M24, M42	S1, S9A, S49, S8/S53*, M16, M18, M20, M24, M42	S1, S9A, S49, S8/S53*, M16, M18, M20, M24, M42	S1, S9A, S49, S8/S53*, M16, M18, M20, M24, M42	S1, S9A, S49, M16, M18, M20, M24, M42	S1, S9A, S49, M18, M20, M24, M42	S1, S9A, M18, M24, M42	S1, S49, M16, M18, M24, M42
Citrate catabolism	CL	CL	CL	CL	CL	CL		CL	CL	CL
Transporters	ABC (sugars, phosphonate, Fe), sugar-phosphate permease, glucose:Na$^+$ symporter, amino acid permease, amino acid/polyamine transporter, uracil permease, PO_4^{3-} transporter (PiT), Fe^{3+} transporter (Feo), Zn transporter (ZupT), Mg/Co transporter	ABC (sugars, phosphonate, Fe), sugar-phosphate permease, glucose:Na$^+$ symporter, uracil permease	ABC (sugars, phosphonate, Fe), glucose:Na$^+$ symporter, amino acid permease, uracil permease, PO_4^{3-} transporter (PiT), Fe^{3+} transporter (Feo), Mg/Co transporter	ABC (sugars, phosphonate, Fe), malto-oligosaccharide permease, amino acid/polyamine transporter, PO_4^{3-} transporter (PiT), Fe^{3+} transporter (Feo), Mg/Co transporter	ABC (sugars, phosphonate, Fe), malto-oligosaccharide permease, amino acid permease, amino acid/polyamine transporter, PO_4^{3-} transporter (PiT), Fe^{3+} transporter (Feo), Zn transporter (ZupT), Mg/Co transporter	ABC (sugars, phosphonate, Fe), malto-oligosaccharide permease, amino acid/polyamine transporter, PO_4^{3-} transporter (PiT), Fe^{3+} transporter (Feo), Zn transporter (ZupT), Mg/Co transporter	ABC (sugars, phosphonate, Zn, Mo), PO_4^{3-} transporter (PiT), Fe^{3+} transporter (Feo), Zn transporter (ZupT), Mg/Co transporter	ABC (sugars, BCAA, phosphonate, Zn, Mo), uracil permease, PO_4^{3-} transporter (PiT), Na$^+$:PO_4^{3-} cotransporter, Fe^{3+} transporter (Feo), Mg/Co transporter	ABC (sugars, BCAA, Fe, Zn), amino acid/polyamine transporter, PO_4^{3-} transporter (PiT), Na$^+$:PO_4^{3-} cotransporter, Mg/Co transporter	ABC (sugars, BCAA, phosphonate, Fe, Zn), uracil permease, PO_4^{3-} transporter (PiT), Na$^+$:PO_4^{3-} cotransporter, Mg/Co transporter
Ammonia assimilation	GS, GDH	GS, GDH	GS, GDH	GS, GDH	GS, GDH	GS, GDH	GS, GDH	GDH	GDH	GS
Oxidative stress	KatA, Dfr	KatA, Dfr	Dfr	Dfr	Dfr	Dfr	Dfr	KatA, Dfr	Dfr	Dfr

ABC, ATP-binding cassette transport system; Ack, acetate kinase; ACL, ATP-citrate lyase; Adh, alcohol dehydrogenase; AOR, aldehyde:ferredoxin oxidoreductase; BCAA, branched-chain amino acids; Buk, butyrate kinase; CL, citrate lyase; CysC, adenylylsulfate kinase; Dfr, desulfoferrodoxin; GH, glycoside hydrolase; GlpK, glycerol kinase; GDH, glutamate dehydrogenase; GS, glutamine synthetase; Hdr, heterodisulfide reductase; HppA, pyrophosphate-energized sodium pump; IOR, indolepyruvate:ferredoxin oxidoreductase; KatA, catalase; Mrp, multicomponent Na$^+$:H$^+$ antiporter; Nqr, sodium-translocating NADH:quinone oxidoreductase; PGA, poly-γ-glutamate; POR, pyruvate:ferredoxin oxidoreductase; Pta, phosphate acetyltransferase; Ptb, phosphate butyryltransferase; Rnf, ferredoxin:NAD$^+$-oxidoreductase complex; rTCA cycle, reverse tricarboxylic acid cycle; Sat, sulfate adenylyltransferase; SCFA, short-chain fatty acids; Sud, bifunctional sulfide dehydrogenase/ferredoxin:NADP oxidoreductase; TFR, thiol:fumarate reductase; TST, thiosulfate:sulfurtransferase (rhodanese-like); TusA, sulfur relay protein; VOR, 2-oxoisovalerate:ferredoxin oxidoreductase. * indicates protein has C-terminal dockerin domain.

Table S6 | Metabolic traits inferred from analysis of Ace Lake Omnitrophota MAGs.

Class (GTDB)	Koll11					4484-49				Koll11				
Order (GTDB)	UBA10015			GIF10	4484-171	4484-49			--	UBA1560		UBA10183	CG1-02-43-210	
Family (GTDB)	--		Kpj58rc	--	4484-171	4484-49	--		--	--		UBA10183	--	
Candidatus genus	Aceula		Zapsychrus	Gygaella	Susulua	Saelkia	Kaelpia		Kappaea	Tantalella	Aadella	Gorgyraea	Orphnella	
Candidatus species	lacicola (type)	meridiana	exili (type)	obscura (type)	stagnicola (type)	tenebricola (type)	aquatica (type)	imicola	frigidicola (type)	remota (type)	gelida (type)	atricola (type)	occulta (type)	
MAG ID	3300035698 _1100 (type)	3300035698 _985 (type)	3300035698 _1555 (type)	3300035698 _1934 (type)	3300035698 _1005 (type)	3300035698 _749 (type)	3300035698 _2000 (type)	3300035698 _1655 (type)	3300035698 _1500 (type)	3300035698 _1097 (type)	3300035698 _848 (type)	3300035698 _32 (type)	3300025586 _21 (type)	3300035698 _104
Genome completeness	92%	92%	89%	85%	82%	91%	93%	92%	76%	93%	91%	92%	85%	61%
Hydrogenases	NiFe Group 3d	NiFe Group 3d	NiFe Group 3d	FeFe Group A3	FeFe Group A3	NiFe Group 4g	FeFe Group A3	FeFe Group A3	FeFe Group A3	NiFe Group 3b / Shy	NiFe Group 3b / Shy	NiFe Group 4g	NiFe Group 3b/ Shy	NiFe Group 3b / Shy
Energy conservation	Rnf, HppA, Sud, Etf	Rnf, HppA, Sud, Etf	Rnf, HppA, Sud, Etf	Rnf, HppA, Sud, Etf	Rnf, HppA, Sud, Etf	Rnf, HppA, Sud, Etf, Mrp	Rnf, HppA, Sud, Etf	Rnf, HppA, Sud, Etf	Rnf, HppA, Etf	Rnf, HppA, Sud, Etf	Rnf, Sud, Etf	Rnf, HppA, Sud, Etf, Mrp, WLP	Rnf, HppA	Rnf, HppA, Sud
Ferredoxin oxidoreductases	POR (mon)	POR (mon)	POR (mon)	POR (multi), VOR	POR (multi), VOR	POR (multi), VOR	POR (multi), VOR	POR (multi), VOR	POR (mon),	POR (mon), VOR	POR (mon), VOR	VOR	VOR	POR (multi), VOR
Citrate synthase	Si	Si	Si	Si	Si	Re	Si	Si	Re	Re	Re	Re	Re	Re
SCFA catabolism				Pta+Ack	Pta+Ack	Pta+Ack	Pta+Ack	Pta+Ack	Pta+Ack	Pta+Ack	Pta+Ack	Pta+Ack		Pta+Ack
Glycerol catabolism	GlpK		GlpK							GlpK	GlpK			
Alcohol catabolism						Adh	Adh	Adh		Adh				
Poly/ oligosaccharide degradation (glycoside hydrolases)	GH2, GH5, GH94/GH36	GH2, GH94/GH36, GH65	GH2, GH5, GH94/36	GH5, GH94/GH36, GH65	GH1, GH2, GH94/GH36, GH65	GH94/GH36, GH65	GH94/GH36, GH65	GH94/GH36, GH65	GH2, GH35, GH94/GH36, GH65	GH1	GH1	GH2, GH94/GH36, GH65	GH2, GH94/GH36, GH65	GH2, GH94/GH36, GH65
Glycogen/α-glucan synthesis	GlgABC	GlgABC	GlgABC	GlgABC	GlgABC	GlgABC	GlgABC	GlgABC		GlgABC		GlgABC		
Polypeptide degradation (proteases/ peptidases)	M17, M24, M48, M50, S1C	M17, M24, M48, M50, S1C	M16, M17, M24, M48, M50, S1C	M24, M48, M50, S1C	M16, M24, M50, S1C	M16, M17, M24, M50, S1C	M16, M17, M24, M50	M16, M17, M24, M50, S1C	M16, M24, M48, M50	M16, M24, M50	M16, M17, M24, M48, M50	M16, M24, M48, M50, S1C	M16, M48, S1C	M16, M24, M48, M50, S1C
Transporters	ABC (sugar, oligopeptide), amino acid permease, PO_4^{3-}:Na^+ symporter, Fe^{3+} transporter (Feo), NH_4^+ transporter (Amt)	ABC (sugar, oligopeptide), PO_4^{3-}:Na^+ symporter, Fe^{3+} transporter (Feo), NH_4^+ transporter (Amt)	ABC (sugar, oligopeptide), amino acid permease, PO_4^{3-}:Na^+ symporter, Fe^{3+} transporter (Feo), NH_4^+ transporter (Amt)	ABC (sugar, oligopeptide, Fe), amino acid permease, PO_4^{3-}:Na^+ symporter, Fe^{3+} transporter (Feo)	ABC (sugar, oligopeptide), PO_4^{3-}:Na^+ symporter, Fe^{3+} transporter (Feo)	ABC (sugar, oligopeptide, Fe), amino acid permease, PO_4^{3-}:Na^+ symporter, Fe^{3+} transporter (Feo)	ABC (sugar, oligopeptide, Fe), amino acid permease, PO_4^{3-}:Na^+ symporter, Fe^{3+} transporter (Feo), NH_4^+ transporter (Amt)	ABC (sugar, oligopeptide, Fe), amino acid permease, PO_4^{3-}:Na^+ symporter, Fe^{3+} transporter (Feo)	ABC (sugar, Fe), sugar-proton symporter, Fe^{3+} transporter (Feo)	ABC (sugar, oligopeptide), sugar-proton symporter, Fe^{3+} transporter (Feo)	ABC (sugar, oligopeptide, Fe, Co/Ni), amino acid permease, Fe^{3+} transporter (Feo), NH_4^+ transporter (Amt)	ABC (sugar, oligopeptide, Fe), PO_4^{3-}:Na^+ symporter, Fe^{3+} transporter (Feo)	ABC (sugar, Fe), Fe^{3+} transporter (Feo)	ABC (oligopeptide, Fe), sugar-proton symporter, PO_4^{3-}:Na^+ symporter
Ammonia assimilation	GS, GOGAT, GDH	GS	GS, GOGAT, GDH	GS	GS, GDH	GS, GDH	GS, GDH	GS, GDH		GS, GDH	GS, GDH	GS, GDH	GS, GDH	GS, GDH
Oxidative stress	Dfr	Dfr	Dfr	Dfr		Dfr	Dfr	Dfr		Dfr	Dfr	Dfr	Dfr	Dfr

ABC, ATP-binding cassette transport system; Ack, acetate kinase; AcsABCD, carbon monoxide dehydrogenase/acetyl-CoA synthase complex; Adh, alcohol dehydrogenase; Dfr, desulfoferrodoxin; Etf, electron transfer flavoprotein; FocA, formate transporter; GH, glycoside hydrolase; GlgABC, glycogen storage proteins; GlpK, glycerol kinase; GDH, glutamate dehydrogenase; GOGAT, glutamate synthase; GS, glutamine synthetase; HppA, pyrophosphate-energized sodium pump; mon, monomeric; multi, multimeric; POR, pyruvate:ferredoxin oxidoreductase; Pta, phosphate acetyltransferase; Rnf, ferredoxin:NAD^+-oxidoreductase complex; SCFA, short-chain fatty acids; Shy, sulfhydrogenase; Sud, bifunctional sulfide dehydrogenase/ferredoxin:NADP oxidoreductase; VOR, 2-oxoisovalerate:ferredoxin oxidoreductase; WLP, Wood-Ljungdahl pathway.
